# Artificial Neural Networks for the Prediction of Monkeypox Outbreak

**DOI:** 10.3390/tropicalmed7120424

**Published:** 2022-12-08

**Authors:** Balakrishnama Manohar, Raja Das

**Affiliations:** Department of Mathematics, School of Advanced Sciences, Vellore Institute of Technology (VIT), Vellore 632014, India

**Keywords:** Hessian matrix, Levenberg–Marquardt model, K-fold cross-validation, regression analysis, machine learning, COVID-19, sigmoid function

## Abstract

While the world is still struggling to recover from the harm caused by the widespread COVID-19 pandemic, the monkeypox virus now poses a new threat of becoming a pandemic. Although it is not as dangerous or infectious as COVID-19, new cases of the disease are nevertheless being reported daily from many countries. In this study, we have used public datasets provided by the European Centre for Disease Prevention and Control for developing a prediction model for the spread of the monkeypox outbreak to and throughout the USA, Germany, the UK, France and Canada. We have used certain effective neural network models for this purpose. The novelty of this study is that a neural network model for a time series monkeypox dataset is developed and compared with LSTM and GRU models using an adaptive moment estimation (ADAM) optimizer. The Levenberg–Marquardt (LM) learning technique is used to develop and validate a single hidden layer artificial neural network (ANN) model. Different ANN model architectures with varying numbers of hidden layer neurons were trained, and the K-fold cross-validation early stopping validation approach was employed to identify the optimum structure with the best generalization potential. In the regression analysis, our ANN model gives a good R-value of almost 99%, the LSTM model gives almost 98% and the GRU model gives almost 98%. These three model fits demonstrated that there was a good agreement between the experimental data and the forecasted values. The results of our experiments show that the ANN model performs better than the other methods on the collected monkeypox dataset in all five countries. To the best of the authors’ knowledge, this is the first report that has used ANN, LSTM and GRU to predict a monkeypox outbreak in all five countries.

## 1. Introduction

Almost all countries in the world were impacted by the COVID-19 pandemic that started in December 2019 in Wuhan, China. The onset of monkeypox in 2022, as reported by several nations, is another concern worldwide. The infectious condition known as monkeypox is brought on by the Zoonotic Ortho-poxvirus, a member of the Poxviridae family and the genus Ortho-poxvirus. It is closely linked to both cowpox and smallpox [1,2]. It is primarily transmitted by monkeys and rodents, although human-to-human transmission is also very common [3,4]. In a Copenhagen, Denmark lab in 1958, the virus was found for the first time in a monkey’s body [5,6]. During a stepped-up drive to eliminate smallpox in 1970, the Democratic Republic of the Congo observed the first instance of monkeypox in a person [7,8]. Monkeypox often affects a large number of people who live close to tropical rainforests in central and western Africa. When a person comes into intimate touch with another infected individual, animal or object, the virus itself spreads. Direct bodily contact, animal bites, respiratory droplets or mucus from the eyes, nose or mouth can all spread the disease [9,10,11]. Fever, bodily pains and exhaustion are a few of the warning symptoms of a monkeypox infection in patients, with a red bump on the skin being the long-term result [12,13]. Although COVID-19 has been found to be more contagious than monkeypox so far, the number of cases is still increasing. Only 50 cases of monkeypox were reported in West and Central Africa in 1990 [14,15]. However, in 2020, there were 5000 instances. In the past, monkeypox was thought to exist solely in Africa, but, in 2022, numerous additional non-African nations in Europe and the United States reported finding cases of the virus [16,17]. As a result, people are gradually becoming more and more anxious and afraid, which is frequently reflected in people’s opinions on social media.

The first monkeypox outbreak was found in the UK on 6 May 2022. At the time of this writing, there are a total of 52,379 cases globally. The top five nations where it is more extreme are the USA (19,355 cases), Germany (3480 cases), the UK (3419 cases), France (3547) and Canada (1286) [18]. Despite the worldwide vaccination efforts, 17 people have died due to the virus, demonstrating that it is not fatal but is very infectious and evolves into other sub-variants. If not treated properly and controlled, the world may continue to see more new instances and deaths.

The Centers for Disease Control and Prevention (CDC) stated that there is currently no completely effective therapy for the monkeypox virus [19,20]. The CDC approved two oral medications, Brin-cidofovir and Tecovirimat, which had mostly been used to treat the smallpox virus but have now been utilized to treat the monkeypox virus in order to meet the urgent demand [21]. Although vaccinations against the monkeypox virus are available and have received FDA approval, they have not yet been used on humans in the United States. The vaccinations for the smallpox virus are used to treat the monkeypox virus in other nations [20].

Regression techniques using ANN methods are commonly used in order to predict future patient issues related to a particular disease [22]. Using the aforementioned methods, several research studies have been carried out to anticipate the range of devastation caused by diseases such as breast cancer, cardiovascular disease and COVID-19. The primary objective of this study is to forecast the confirmed cases of monkeypox in real-time. It also examines the early surveillance and prediction of the monkeypox pandemic [23]. Such real-time prediction systems could be very helpful for healthcare professionals and government agencies in guiding early responses for the very successful and timely management of this diseases. The decisions to control current possibilities can be made using these systems.

The actual number of monkeypox infection data indicates a set of observations that have been chronologically ordered. Time-series prediction techniques originated in statistics. Machine learning-based techniques, meta-predictors, structure-based techniques and ANNs also exist for this purpose [14,15]. ANNs are frequently employed for time series predictions [24]. One of the main benefits of an ANN approach is that it may be fed with unprocessed data that can automatically identify the necessary representation [25]. The ANN produces trustworthy outcomes based on a number of variables, including performance, accuracy, latency, speed, convergence and size. The USA, Germany, the UK, France and Canada are five of the nations with the highest number of monkeypox illnesses. No extensive works have been done on the monkeypox outbreak to the best of our knowledge.

The main contribution of this paper is that we have developed a forecasting model of the monkeypox time series dataset in the five countries mentioned above by employing state-of-the-art ANN models such as the LSTM and GRU. This study uses the ANN with an LM optimizer and the LSTM and GRU uses an ADAM optimizer. It further predicts the number of monkeypox cases directly resulting from this disease using NNs. These NNs used the existing datasets that contained all the available data related to the monkeypox epidemic in countries such as the USA, Germany, the UK, France and Canada.

The comparative study of these five countries will help the healthcare authorities to prepare for the necessary actions that need to be taken based on our model predictions. A summary of monkeypox from 6 May 2022 to 24 August 2022 in the USA, Germany, the UK, France and Canada has been considered.

The rest of the paper is organized as follows. The methods and materials are shown in Section 2, followed by a description of the ANN, LSTM and GRU structures, along with the LM and ADAM optimizers. The results are presented and analyzed in Section 3. We come to certain conclusions in Section 4.

### Related Works

Many researchers, including data scientists, have expended a lot of efforts to forecast the spread of this disease. By creating prediction models that emphasize the likely behaviors of this virus, data scientists may significantly advance the knowledge by improving our ability to forecast how the virus will spread. As a result, ANN models are thought of as precise tools that may aid in creating prediction models. In fact, several neural networks (NNs) have been developed in the past [26], as shown in Table 1.

## 2. Methods and Materials

### 2.1. Artificial Neural Network

The ANN is a simple imitation of the neuron structure of the human brain [34]. Basic scalar messages, simple processing components, a high degree of interconnection and adoptive interaction between the units are the things which make them a type of multi-processor computer system [35]. Actually, the ANN provides a reasonably quick and flexible way of modelling, so it is appropriate for a rainfall-runoff prediction [36]. Layers of neurons make up an ANN. One or more hidden layers of neurons connect the input layer of neurons to the output layer of neurons. The interconnecting link between the neuron layers is made up of connection weights. This method changes its weights throughout the training phase to reduce the errors between the projected result and the actual output using the Back Propagation algorithm [37]. To get the best topology and weights, an ANN is trained using experimental data (called training data) and then evaluated with more experimental data (test-data). The accuracy of the model is checked using validation data. Bias refers to the weight that is provided directly to one neuron without being coupled to the prior neuron in specific circumstances. The most common type of ANN is the multilayer perceptron (MLP). It also has one or more hidden layers in the feed forward neural network.

A data structure that may operate like a neuron is constructed in ANNs with the use of programming skills. A node is a kind of data structure [38,39]. The network connecting these nodes is trained in this structure using a standard training technique like gradient descend. The nodes have two active states (on or off) and one inactive state (off or 0) in this memory or neural network, and each edge (synapse or link between nodes) has a weight. Negative weights inactivate or inhibit the following linked node (if active), whereas positive weights stimulate or activate the next inactive node [40,41,42,43]. The input *d_p_* reaches the neuron *c* from the preceding neuron p in the ANN architecture. *T_c_* is the total of the products of the inputs and their weights from Equation (1) [44], and *w_pc_* is the weight of the input *d_p_* with regard to cell *c*.

(1) $$T_{c}\;=\;\sum w_{pc}d_{p}$$

The sigmoid function was chosen as the activation function and is applied to *T_c_*. As a result, Equation (2) [44] is used to compute *d_c_*:

(2) $$d_{c}\;=\;sigmoid_{c}\;\left(T_{c}\right)$$

Likewise, the weights of the *d_cn_* are *w_cn_*, which is the output of *c* to *n*, which are computed. In a set, *W* is the sum of all the weights of the neural network, and kw(x) is the neural network’s output for input x and output y. The key purpose is to figure out these weights so that the error values between y and kw (x) are reduced. That is, the aim is to reduce the cost function *E*(*W*) (Equation (3)) to the smallest possible value [44]:

(3) $$E\left(w\right)\;=\;\frac{1}{2}{\sum_{i=1}^{n}\left(y_{i}-o_{i}\right)}^{2}\;$$

**Figure 1:**
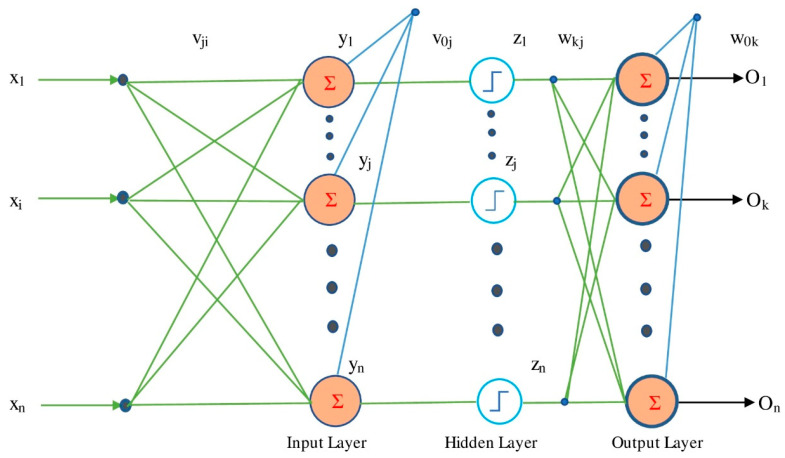
Architecture of an artificial neural network.

In this study, the ANN with LM optimizer [38], one of the most commonly used varieties of ANN, was utilized to forecast the epidemic. ANN was trained on a dataset using the LM algorithm. The optimum response was achieved by training the network with selected inner neurons. To minimize the cost function value, the results were calculated using the RMSE and correlation coefficient. The ANNs architecture is shown in Figure 1.

### 2.2. Levenberg–Marquardt Algorithm (LM)

The LM approach applies another approximation to the Hessian matrix in order to ensure that the estimated Hessian matrix *J^T^J* is invertible [45].

(4) $$H\;\approx \;J^{T}J\;+\;\mu \;I\;$$

Here, *μ* is called the combination coefficient and is always positive and *I* is the identity matrix.

The components on the basic diagonal of the estimated Hessian matrix will be bigger than zero, as shown in Equation (5). As a result of this approximation (Equation (5)), the invertibility of matrix *H* can be guaranteed [46].

The update rule of the LM algorithm could be represented by merging Equations (4) and (5) as follows.

(5) $$V_{k+1}=\;v_{k}-\;{\left(J_{k}^{T}J_{k}+\;\mu I\right)}^{-1}\;J_{k}e_{k}$$

Here, the weight vector is *V* and the error vector is *e_k_*.

**Figure 2:**
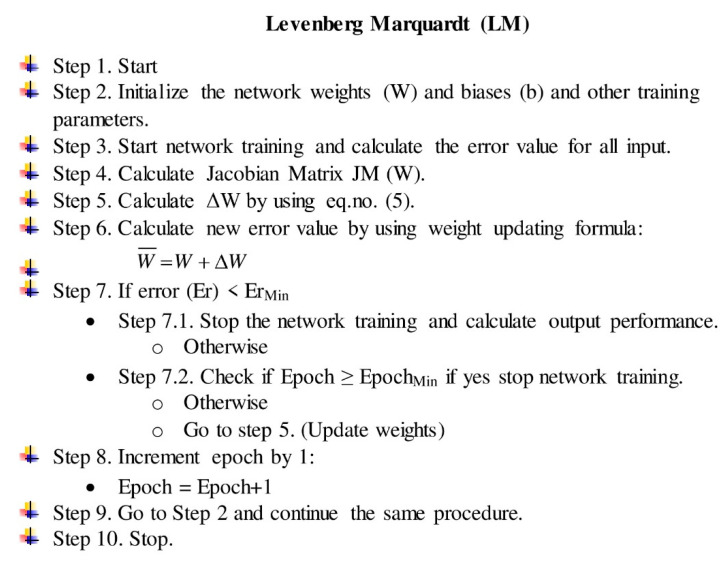
Stepwise Levenberg–Marquardt algorithm procedure.

The LM algorithm shifts between the two techniques throughout the training phase, using a mix of the steepest descent algorithm and the Gauss–Newton algorithm. Equation (5) is the Gauss–Newton procedure, which is utilized when the combination coefficient is very tiny [47] (almost 0). Equation (5) approximates Equation (4), and the steepest descent approach is applied when the combination coefficient is extremely big. We explain the stepwise procedure of the LM algorithm in Figure 2.

### 2.3. Long Short-Term Memory (LSTM)

**Figure 3:**
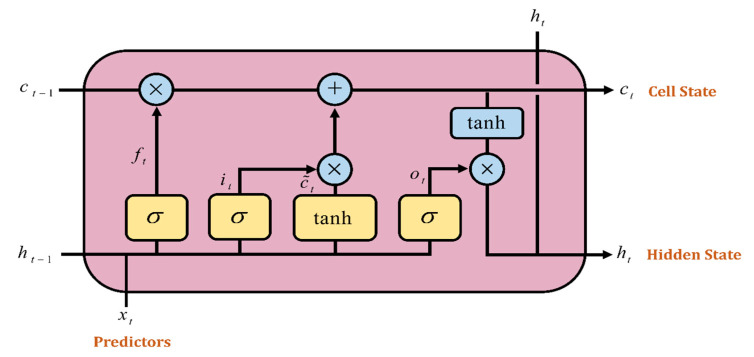
Long Short-Term Memory (LSTM) gates.

The LSTM networks are made up of different gates that store information about the prior state. These data are either written, saved or retrieved from a cell that functions as a memory. When the cell reads, writes and erases using the gates that open and close, it determines whether to save the incoming information. They act depending on the signals they receive, blocking or passing on information according to its strength and import by filtering with their own weights. These weights are similar to those used to regulate the input and hidden states via the network’s training process [48]. This study proposes a LSTM network with an input layer, a hidden layer and an output layer. Figure 3 depicts an LSTM model with an input gate *i_t_*, output gate *o_t_*, forget gate *f_t_* and cell state, *c_t_*.

Memory blocks, which were designed to deal with disappearing gradients by remembering network parameters for long periods of time, are the most basic components of LSTM networks. Memory blocks in the LSTM architecture are analogous to digital systems’ differential storage structures [49]. The activation functions (sigmoid and tansig) used by the gates in LSTM aids in the processing of information, and the output is either 0 or 1. Because we need to transfer only positive values to the following gates in order to produce a clear output, we employ sigmoid and tansig as activation functions [50]. The following Equations (6)–(11) represent the three gates of the LSTM network:

(6) $$f_{t}\;=\;\sigma \left(x_{t}W_{f}+h_{t-1}u_{f}+b_{f}\right)\;\;$$

(7) $$i_{t}\;=\;\sigma \left(x_{t}W_{i}+h_{t-1}u_{i}+b_{i}\right)\;\;$$

(8) $$o_{t}\;=\;\sigma \left(x_{t}W_{o}+h_{t-1}u_{o}+b_{o}\right)\;\;\;$$

(9) $${\bar{C}}_{t}\;=\;\tanh\left(x_{t}W_{C}+h_{t-1}u_{C}+b_{C}\right)\;$$

(10) $$C_{t}\;=\;\sigma \left(f_{t}+C_{t-1}+i_{t}+\bar{C_{t}}\right)\;$$

(11) $$h_{t}\;=\;\tanh\left(C_{t}\right)\;\times \;o_{t}$$

Matrices *W_q_* and *u_q_* contain the weights of the input and recurrent connections, where the index can be the input gate i, output gate o, the forgetting gate f or the memory cell c, depending on the activation being calculated. *C_t_* is not just a cell of an LSTM unit, but contains h cells of the LSTM units, while *i_t_*, *o_t_* and *f_t_* represent the activations of, respectively, the input, output and forget gates, at time step t, where:

*i_t_* = input gate function;

*f_t_* = forget gate function;

*o_t_* = output gate function;

*h_t_* = hidden state function, also known as the output gate of the LSTM unit;

$\bar{C_{t}}$ = cell input activation state;

*C_t_* = cell state vector;

*h*_t−1_ = the result from the prior time step;

where *W*, *u* and *b* are the weight matrices and bias vector parameters which need to be learned during the training.

### 2.4. Gated Recurrent Unit (GRU)

The GRU is one of the variants of the RNN which was introduced by Cho et al. [28]. The update gate and resets gate are the two gates that the GRU utilizes. These gates employ activation functions similarly to the LSTM. The information from earlier time steps is added to the input data for time step t before being delivered to the update gate. This gate determines how much of this data flow needs to be sent on to the future and functions similarly to how the input gate and forget gate combine in the LSTM network. The reset gate determines how much of the previously computed state should be forgotten and stores the necessary data. The update gate determines what to collect from the previous steps and the current memory content to calculate the output of the current unit [49,51].

**Figure 4:**
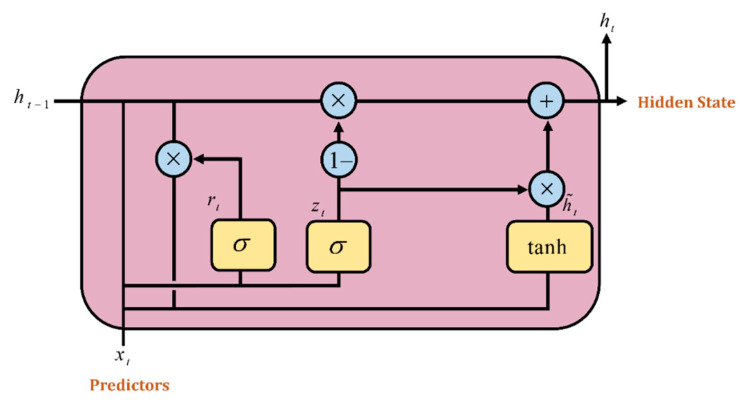
Gated Recurrent Unit (GRU) gates.

Figure 4 shows the internal architecture of a GRU unit cell. The mathematical Equations (12)–(15) are used to calculate these respective gates:

(12) $$z_{t}\;=\;\sigma \left(x_{t}W^{z}+h_{t-1}u^{z}+b_{z}\right)\;\;\;\;\;\;$$

(13) $$r_{t}\;=\;\sigma \left(x_{t}W^{r}+h_{t-1}u^{r}+b_{r}\right)$$

(14) $${\bar{h}}_{t}\;=\;\tanh\left(r_{t}\times h_{t-1}u+x_{t}W+b\right)\;$$

(15) $$h_{t}\;=\;\left(1-z_{t}\right)\times \;\;{\bar{h}}_{t}\;+\;\;z_{t}\times h_{t-1}$$

where *W_z_*, *W_r_* and *W* denote the weight matrices for the corresponding connected input vector. *u_z_*, *u_r_* and *u* represent the weight matrices of the previous time step and *b_r_*, *b_z_* and *b* are the bias. The *σ* denotes the logistic sigmoid function, *r_t_* denotes the reset gate, *z_t_* denotes the update gate and $\bar{h}$*_t_* denotes the candidate hidden layer.

Deep learning networks are very sensitive to hyperparameters. The forecasted output will oscillate at high frequencies when the hyperparameters are wrongly configured [52]. The number of hidden neurons in the recurrent layers, the number of dropouts and the value of the learning rate are essential hyperparameters for GRU network models.

### 2.5. Adaptive Moment Estimation Optimization (ADAM)

Classification can be difficult when dealing with problems relating to the learning process. Several approaches have been proposed to help us arrive at an optimal learning level. The ADAM optimization algorithm is a deep learning extension of the stochastic gradient descent algorithm, which has recently been used in a variety of applications on the Internet of Things (IoT), text detection and so on [53].

ADAM is a famous optimizer that combines a gradient descent with momentum and the RMSprop optimizer [54]. The weights are updated using:

(16) $$\theta_{t}\;=\;\theta_{t-1}\;-\left(\alpha /\sqrt{v_{t}^{\prime }+\varepsilon }\right)\times m_{t}^{\prime }\;$$

where $v_{t}^{\prime }$ and $m_{t}^{\prime }$ are the bias correction for the first and second moments, respectively:

(17) $$m_{t}^{\prime }\;=\;m_{t}/\left(1-\beta_{1}^{t}\right)\;\;$$

(18) $$v_{t}^{\prime }\;=\;v_{t}/\left(1-\beta_{2}^{t}\right)\;$$

(19) $$m_{t}\;=\;\beta_{1}m_{t-1}+\;\left(1-\beta_{1}\right)\times \;g_{t}\;$$

(20) $$v_{t}\;=\;\beta_{2}v_{t-1}+\;\left(1-\beta_{2}\right)\times \;g_{t}^{2}$$

In Equation (19), *m_t_* is the first moment that represents the running average of the gradients, whereas in Equation (20), *v_t_* is the second moment that represents the running average of the squared gradients.

### 2.6. Network Modelling Process

The NN modelling procedure was carried out in two stages, including training and testing. The data must fall inside a narrow range to hasten the model convergence and improve the forecast accuracy. The study’s input data were either in the tens of thousands or single digits. As a result, the min–max approach (Equation (21)), which requires that all input data points fall inside the range [0,1], was used using the following transformation.

(21) $$X_{i,j}\;=\;\frac{X_{i}-X_{i,\min}}{X_{i,\max}-X_{i,\min}}\;\;\;\;\;$$

When “*x_i, j_*” refers to “*x_i_*”, the actual value of the input variable *‘i’* is normalized. The minimum and maximum values of the input variable ‘*i*’ are, respectively, “*x_i, min_*” and *“x_i, max_”.* Similar to this, Equation (21) was used to normalize the target values so that they would fall inside the [0,1] operating range of the activation function.

**Figure 5:**
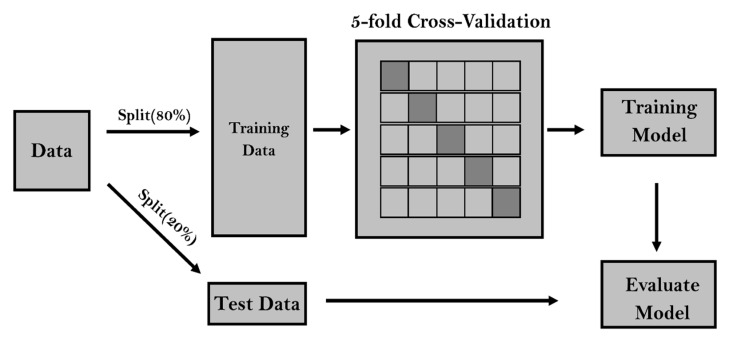
The flowchart of the research methodology workflow.

The complete dataset was divided into two separate subsets after the data normalization. The workflow of the research methodology is shown in Figure 5.

(1) Training dataset: in order to reduce the error function, the model’s synaptic weights were adjusted to correspond to the perfect number of hidden layer neurons. The cross-validation method was used to further split the training dataset into “K” subsets in order to find the ideal number of iterations (or “epochs”) before the model training should be terminated.
(2) Testing dataset: following the training phase, it was used to evaluate the model’s accuracy and forecasting capability.

### 2.7. Data Preparation

**Figure 6:**
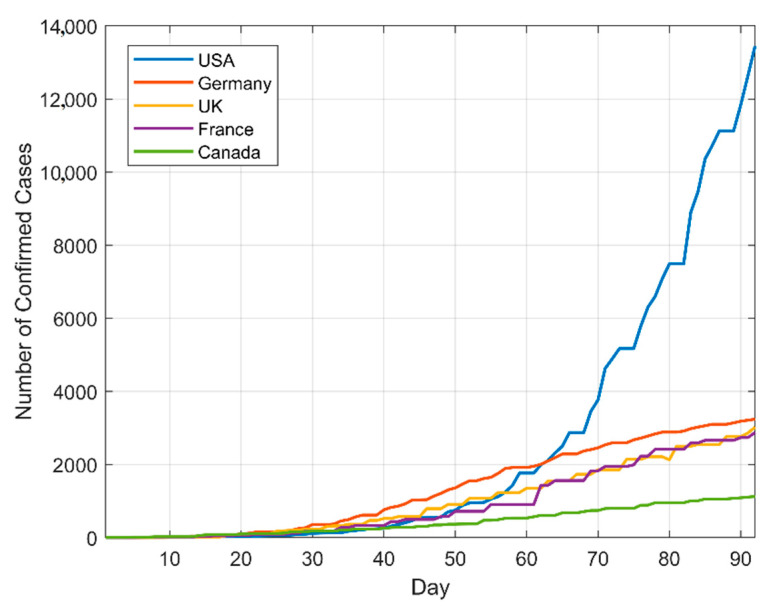
Daily confirmed cases for the USA, Germany, the UK, France and Canada—Monkeypox.

This study used daily confirmed cases data from the USA, Germany, the UK, France and Canada that are available from the “Global. Health” team website. First off, the researchers used data from 6 May 2022—the first case reported—to 31 August 2022 [55]. It was 2.5 MB in size and included 100 records, which is shown in Table 2. The following split of the data for that time period was utilized to discover the right parameters for the models: 80% for training and 20% for testing. Testing was the next stage after training. Figure 6 presents the trend of the daily confirmed cases of monkeypox for the five nations, which include the aforementioned countries.

### 2.8. Netwok Model Evaluation

NN model training is an iterative procedure through which the model learns the input–output behavior. The LM learning method (Equations (4) and (5)) was utilized during the training stage. Two statistical indices were used to evaluate the performance of the model: the coefficient of determination (*R*^2^), which is a measure of the model’s goodness-of-fit, and the root mean squared error (RMSE), which represents the square root of the average squared differences between the target value and the model output value. These two statistical indices are defined by Equations (22) and (23), respectively. The better the model fits the data, the *R*^2^ is nearer to unity and the RMSE value is lower (nearer to zero). In other words, when *R*^2^ equals 1.0 and RMSE equals 0, the model completely fits the data.

(22) R2 = 1−∑i=1nYi^−Yi2∑i=1nYi−Yi¯2    

(23) RMSE = ∑i=1nYi^−Yi2n  

where *Y^^^* represents the predicted values and *Y* represents the actual values, and $\bar{Y}$ represents the mean of the all the values and n denotes the number of values.

### 2.9. K-Fold Cross-Validation

The issue of when to end the training stage of an ANN model is a major conundrum since an overtrained model may perform poorly on an unknown dataset because it has learned the noise instead of the signals. One of the most popular techniques to prevent the model from overtraining is the stop-training criteria based on the k-fold cross-validation [56,57]. The k-fold cross-validation begins with the data being divided into K groups at random, after which the subsequent processes are carried out for each group.

(1) Each fold in the “K” disjoint fold partition of the training dataset has the same number of samples.
(2) In each of the “K” iterations, the model has trained on the first (K-1) folds.
(3) The trained model is subsequently assessed on the final fold (also known as the validation fold) in order to calculate its RMSE.
(4) The number of epochs versus the average RMSE is displayed on the validation folds.

The averaged RMSE typically falls during the early training phase and continues to rise after the network starts over-fitting the data. The RMSE should cease declining while the number of epochs rises, so that the training phase can be terminated.

### 2.10. Network Model Testing

After the model training stage was finished, the trained model was evaluated against the test dataset (which was hidden throughout the training phase) to determine the model’s capacity for prediction. It should be noted that the output values are anti-normalized to their true values once the model training and testing phases are finished.

## 3. Results and Discussion

**Figure 7:**
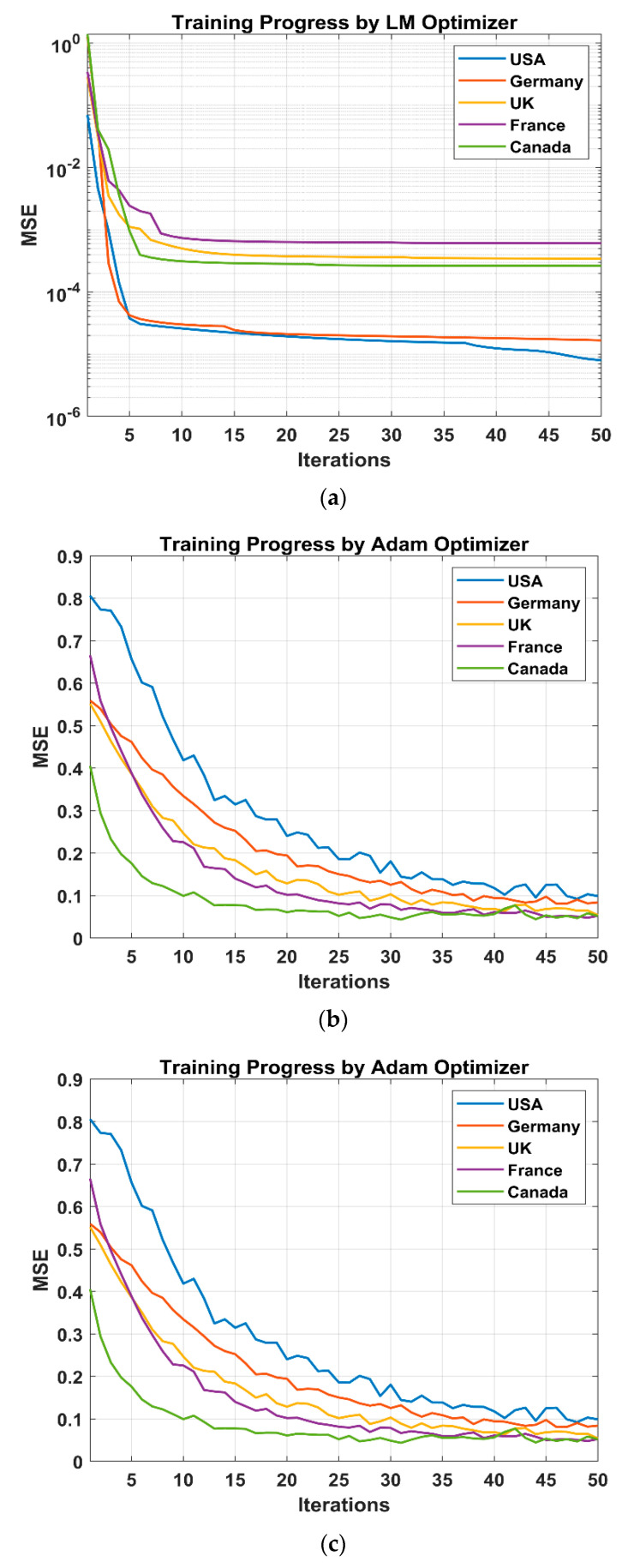
The performance plot of NN training by optimizer mean squared error (MSE) vs. iterations-monkeypox. (**a**) ANN; (**b**) LSTM; (**c**) GRU.

We analyzed the prediction performance of the three neural network models (ANN, LSTM and GRU) on data from five countries, namely the USA, Germany, the UK, France and Canada. The model performances are trained on data from 6 May to 9 August 2022 and evaluated using the test data from 10 August to 31 August 2021. The prediction performance of the models on the test data for all the models is shown in Figure 7.

Before beginning the hybrid modelling process, firstly a perceptron ANN with a single hidden layer and two hidden layers are developed [58,59]. For a sophisticated nonlinear issue, one or two hidden layers will be sufficient to train the ANN [60,61]. In addition, the Levenberg–Marquardt (LM) algorithm is used for the network training. The LM method has been shown to be one of the best and most flexible training algorithms, and as it avoids computing the Hessian Matrix, it could be viewed as the fastest backpropagation technique [15,58,62]. The standard approach described in the literature [63,64] is used to figure out the appropriate number of hidden neurons. In this regard, 1 to 24 ANN models (that means different hidden layers) are developed as shown in Table 3. Each model is categorized according to R^2^ and the RMSE as a result of choosing the best option. A higher number is preferred for R^2^. As a result, under this ranking method, the model with the highest R^2^ obtains the highest score (i.e., the maximum score is 24). On the other hand, a smaller RMSE number will be suitable. Therefore, the model with the lowest RMSE value receives the highest ranking. Moreover, for each model, the overall rank is calculated by adding the two statistics for the training, validation and test stages, independently. Accordingly, in Table 3 the overall ranks attributable to the simulated models are calculated. As can be regarded, model no. 20 with 20 neurons has acquired the maximum total score. It can be claimed that the R^2^ (RMSE) in this model reaches its maximum (minimum) in the training stage. From this point on, the R^2^ will decrease as the number of neurons increases. Subsequently, based on the overall score, this model is picked as the optimal simulation.

In Table 3, we have developed the ANN with a single hidden layer. Based on the highest score, the best architecture of the neural network has been decided for the USA dataset. As per Table 3, we have developed the ANN with two hidden layers (in Table 4) and the models are presented based on the top five highest score. As a result, in Table 3 and Table 4, the best architecture of the neural network has been decided for the USA dataset. For Germany, the UK, France and Canada, we present the top five highest score models in Table 5, Table 6, Table 7 and Table 8, respectively.

Table 3, Table 4, Table 5, Table 6, Table 7 and Table 8 were compared, and we found that single hidden layers performed better than two hidden layers. Therefore, based on the ranking and overall score, the perfect ANN structure was determined for each country using ANN-LM models, i.e., for the USA 5-20-1 (5 neurons in the input layer, 20 neurons in the Hidden layer and 1 neuron in the output layer), for Germany (5-24-1), for the UK (5-18-1), for France (5-5-1) and for Canada (5-16-1).

After acquiring the ANN model’s ideal structure for all the countries’, i.e., 5-20-1 (the USA), 5-24-1 (Germany), 5-18-1 (the UK), 5-5-1 (France) and 5-16-1 (Canada) topology, it was then determined whether the model had been successfully trained or whether an undertraining or overtraining had taken place. A poor training performance is caused by undertraining, and the generalizability of the model might sometimes suffer from overtraining. In other words, the number of epochs at which the training phase is interrupted affects the model’s performance and capacity to generalize. A five-fold cross-validation procedure was used on the training dataset to determine when it is optimal to cease the ANN model’s training. The training MSE curve and the validation MSE curve, both calculated by the five-fold cross-validation, are shown in Figure 7a as functions of the number of training iterations.

### 3.1. Observing the Monkeypox Outbreak Using the ANN-LM Models in the Five Countries

The training performance of an ANN with an LM optimizer is shown in Figure 7a for all five countries. It is clear that at iteration four, the MSE drops drastically to its lowest level, after which the error essentially stays the same. With the best achievement being 0.00001 at iteration 14, the training continues until iteration 50. It is demonstrated that while the adjustment procedure is extremely slow, the LM optimizer always converges extremely quickly by observing all the training processes of an ANN with LM.

**Figure 8:**
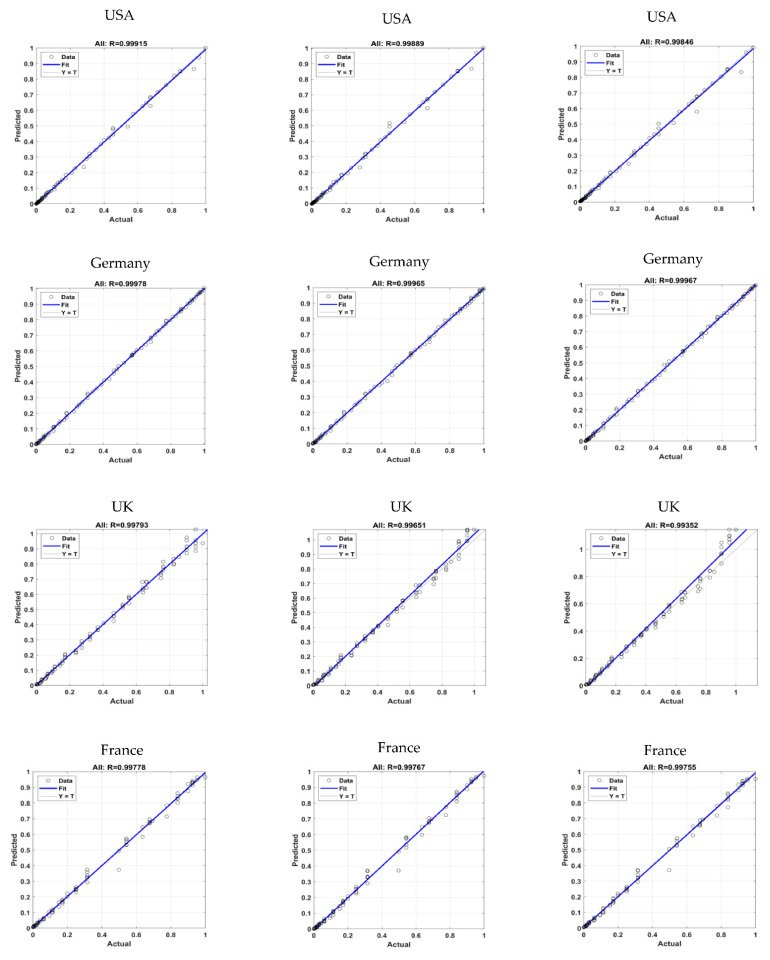

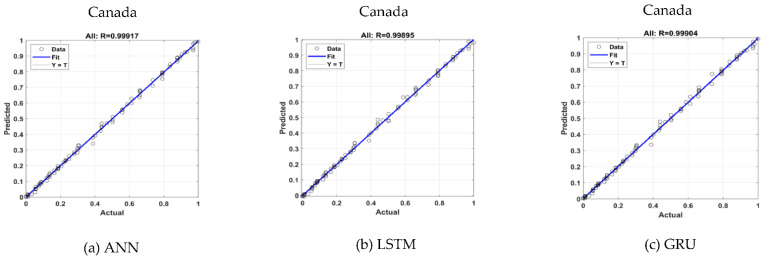
The proposed regression analysis (actual vs. predicted) of neural networks optimal models in predicting—monkeypox.

Regression plots are used to validate the network’s performance, that show the network’s output in terms of targets for training, validation, testing and overall datasets. The entire validation dataset is used by the ANN for training as well. Generally, in a regression plot, if the R-value is nearly 1, that means that the model is perfect (Figure 8a). As the R-value is 0.999 or above in each country, we can see that the fit is quite good (R-value) for the USA, Germany, the UK, France and Canada (0.99915, 0.99978, 0.99793, 0.99778 and 0.99917). Only the ANN-LM algorithm gives the best R-value of almost 0.99999 on the monkeypox outbreak.

**Figure 9:**
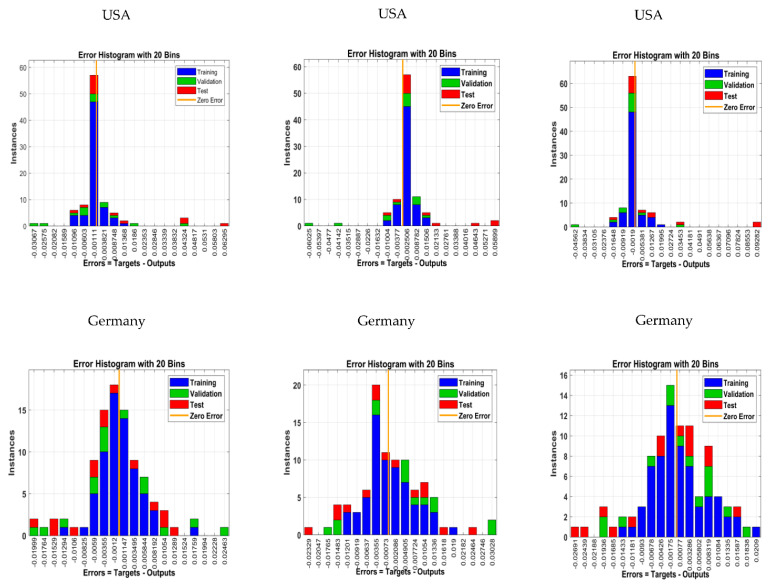

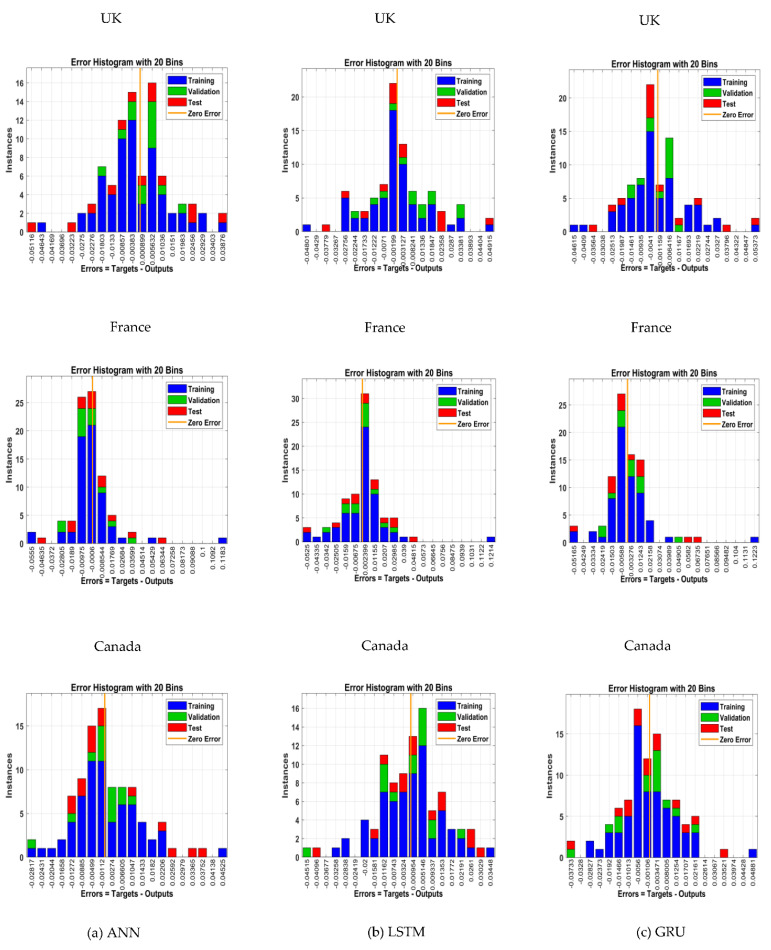
The error histogram of NN optimal model in predicting—monkeypox.

The error histogram is the histogram of the errors between the target values and predicted values after training a neural network. These can be negative as these error values indicate how predicted values differ from the target values. For the ANN-LM model, Figure 9a shows the training data as blue bars, testing data as red bars and validation data as green bars. The graphs are created using the error range (maximum negative error to maximum positive error), which is divided into 20 bins. The histogram makes it possible to spot outliers, which are data points where the fit is noticeably worse than the majority of the data. For the USA, Germany, the UK, France and Canada, Figure 9a shows that more errors in this instance are between −0.00111 and 0.00111, −0.0012 and 0.0012, −0.00383 and 0.00383, −0.0006 and 0.0006 and −0.00112 and 0.00112, respectively. However, there is one learning point (zero line) with 0.003851, 0.001147, 0.000899, 0.008544 and 0.00274 errors, respectively. In this case, we can see that the ANN-LM method gives better results on a monkeypox outbreak.

**Figure 10:**
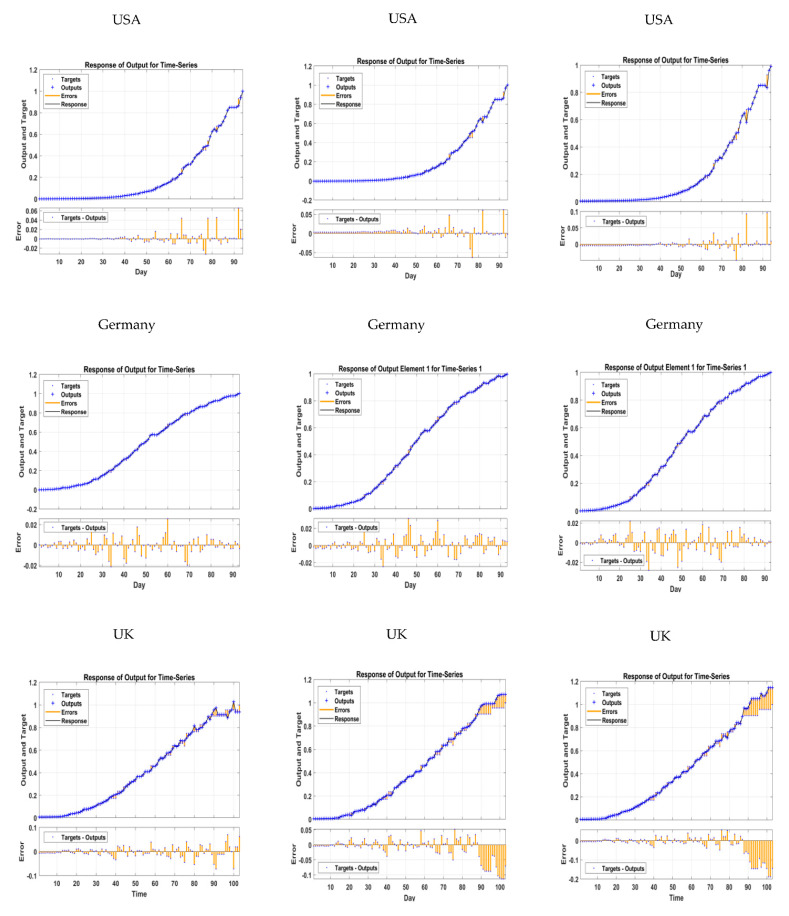

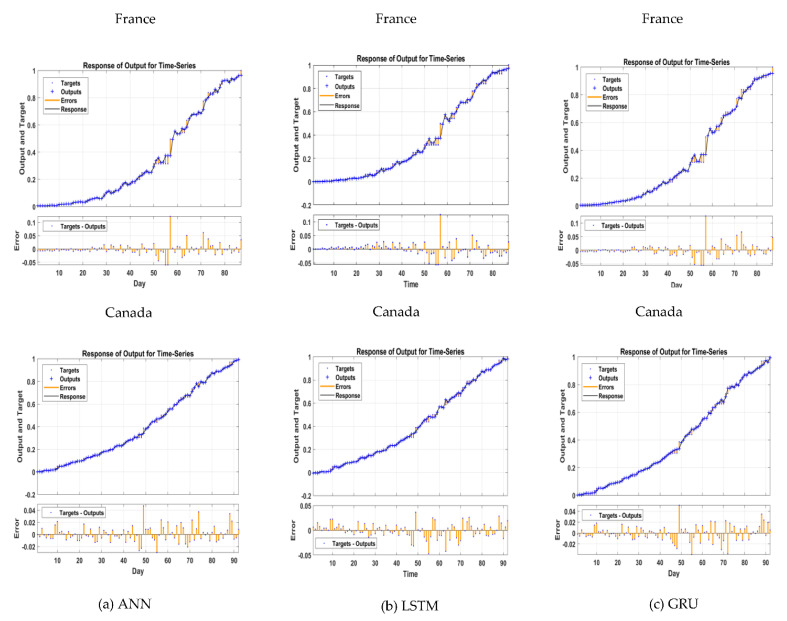
The responds plot of NN optimal model in predicting—Monkeypox.

In Figure 10a, the predicted and actual monkeypox incidence time trends are compared for the model’s performance and accuracy. Plots that compared the observed (target) values to the model-calculated (output) values against time allowed us to observe how the network, outputs and targets responded to the inputs. Additionally, displayed are the errors that were discovered during the process. The ANN-LM model is capable of representing and simulating the desired output, and it provides a good representation of the overall trend of a monkeypox incidence. In addition, the majority of the estimation errors with respect to the time were between −0.02 and 0.02 for all five countries. The results indicate that our model selection was reasonably good.

### 3.2. Observing the Monkeypox Outbreak Using the LSTM-ADAM Models in the Five Countries

The training performance of an LSTM with an ADAM optimizer is shown in Figure 7b for all five countries in same figure. It is clear that at iteration 10, the MSE drops dramatically to its lowest level, after which the error essentially stays the same. With the best achievement being 0.1 at iteration 20, training continues until Iteration 50. It is demonstrated that while the adjustment procedure is extremely slow, the ADAM optimizer always converges extremely quickly by observing all the training processes of an LSTM with ADAM.

As the R-values are (0.99889, 0.99965, 0.99651, 0.99767 and 0.99895), we can see that the fit is quite good (Figure 8b) for all five countries. The LSTM-ADAM model gives the better R-value of almost 0.9888 on the monkeypox outbreak.

For the five countries, Figure 9b shows that more errors are between −0.00377 and 0.00377, −0.00073 and 0.00073, −0.00199 and 0.00199, −0.00675 and 0.00675 and −0.00324 and 0.00324, respectively. However, there is one learning point (zero line) with a 0.002506, 0.002086, 0.003127, 0.002399 and 0.000954 error, respectively. In this case, we can see that the LSTM-ADAM method provides better results on a monkeypox outbreak.

In Figure 10b, the predicted and actual monkeypox incidence time trends are compared for the model’s performance and accuracy. The LSTM-ADAM model is capable of representing and simulating the desired output, and it provides a good representation of the overall trend of a monkeypox incidence. In addition, the majority of the estimation errors with respect to time were between −0.05 and 0.05 for all five countries. We conclude that our model selection was reasonable.

### 3.3. Observing the Monkeypox Outbreak Using the GRU-ADAM Models in the Five Countries

The training performance of GRU with an ADAM optimizer is shown in Figure 7c for all five countries. It is clear that at iteration 15, the MSE drops dramatically to its lowest level, after which the error essentially stays the same. With the best achievement being 0.1 at iteration 25, training continues until iteration 50. It is demonstrated that while the adjustment procedure is extremely slow, the ADAM optimizer always converges extremely quickly by observing all the training processes of GRU with ADAM.

As the R-values are (0.99846, 0.99967, 0.99352, 0.99755 and 0.99904), we can see that the fit is quite good (Figure 8c) for all five countries. The GRU-ADAM model gives the better R-value of almost 0.9888 on the monkeypox outbreak.

For the GRU-ADAM model, Figure 9c shows that more errors in this instance are between −0.0019 and 0.0019, −0.00175 and 0.00175, −0.0041 and 0.0041, −0.00588 and 0.00588 and −0.00106 and 0.00106, respectively. However, there is one learning point (zero line) with a 0.005381, 0.00077, 0.001159, 0.003276 and 0.003471 error, respectively. In this case, we can see that the GRU-ADAM method provides better results on a monkeypox outbreak.

In Figure 10c, the predicted and actual monkeypox incidence time trends are compared for the model’s performance and accuracy. The GRU-ADAM model is capable of representing and simulating the desired output, and it provides a good representation of the overall trend of a monkeypox incidence. In addition, the majority of the estimation errors with respect to the time were between −0.05 and 0.05 for all five countries. We conclude that our model selection was reasonable as a result.

## 4. Conclusions

The monkeypox epidemic has significantly impacted the lives of many people in several nations. This epidemic is becoming worse in certain places. There is currently no treatment for this infection, and there is little chance of accurately forecasting how severe it could be. So, in order to forecast this disease, we designed a neural network model using a time series monkeypox dataset and compared it with the LSTM and GRU models. We used the time series datasets, gathered from the five nations (the USA, Germany, the UK, France and Canada) impacted mostly by monkeypox. The LM learning technique was used to develop and validate a single hidden layer ANN model. Different ANN model architectures with varying numbers of hidden layer neurons were trained, and the K-fold cross-validation early stopping validation approach was employed to identify the optimum structure with the best generalization potential. In the regression analysis, the ANN-LM model gives a good R-value of almost 99%, the LSTM model gives almost 98% and the GRU model gives almost 98%. These three model fittings demonstrated that there was a good agreement between the experimental data and the forecasted values. The results of our experiments show that the ANN model performed better than the other methods on the collected monkeypox dataset in all five countries.

## Figures and Tables

**Table 1:** Summary of related work.

Study	Year	Technique	Input	Output	Results
A COVID-19 time series forecasting model based on MLP ANN [27]	2021	MLP and ANN	Daily confirmed cases	Next 20 days	More than 90%
Deep learning methods for forecasting COVID-19 time-series data: A comparative study [28]	2020	RNN, LSTM, Bi-LSTM and GRUs algorithms	Daily confirmed and recovered cases collected from six countries namely Italy, Spain, France, China, USA and Australia.	Forecasting of the number of new contaminated and recovered cases	VAE achieved MAPE values of 5.90%, 2.19%, 1.88%, 0.128%, 0.236% and 2.04%, respectively
Artificial Neural Networks for Prediction of COVID-19 in Saudi Arabia [24]	2021	ANN and MLPNN–PPA	Confirmed cases and deaths	The number of infected persons will increase in the coming days	The number of recoveries will be 2000 to 4000 per day.
Using Artificial Neural Network with Prey Predator Algorithm for Prediction of the COVID-19: the Case of Brazil and Mexico [29]	2021	ANN, PPA-BMLPNN and PPA-MMLPNN	Confirmed cases, recovered cases and deaths	The number of infected persons will increase in the coming days	The average active cases of COVID-19 in Brazil will go to 9 × 10^5^, with 1.5 × 10^5^ recovered cases per day, and more than 6 × 105 as the total deaths.
Application of artificial neural networks to predict the COVID-19 outbreak [30]	2020	ANN-LM	Daily confirmed cases	The ANN-based model that takes into account the previous 14 days outperforms the other ones	The previous fourteen days for prediction are suggested to predict daily confirmed cases.
Predictions for COVID-19 with deep learning models of LSTM, GRU and Bi-LSTM [31]	2020	ARIMA, SVR, LSTM and Bi-LSTM	Daily confirmed cases and deaths	Prediction of confirmed cases and deaths	Bi-LSTM generates lowest MAE and RMSE values of 0.0070 and 0.0077, respectively
Time series prediction for the epidemic trends of COVID-19 using the improved LSTM deep learning method: Case studies in Russia, Peru and Iran [32].	2020	LSTM	Daily confirmed cases	Next 30 days	The proposed method can accurately analyze the trend of the epidemic.
Artificial neural networks for prediction of COVID-19 in India by using backpropagation [33]	2022	ANN-BP	Daily confirmed cases	The ANN-based model that takes into account the previous 14 days outperforms the other ones	The previous fourteen days for prediction are suggested to predict daily confirmed cases.
Monkeypox	Present	ANN-LM, LSTM-ADAM, GRU-ADAM	Daily confirmed cases	The number of infected persons will increase in the coming days	ANN-LM model (99%) perform better than LSTM and GRU (98%).

**Table 2:** Data description.

Country	Data Description	Country-Code	WHO Region
United States	18 May 2022 to 24 August 2022	USA	Region of the Americas (AMR)
Germany	19 May 2022 to 24 August 2022	DE	European of Region (EUR)
United Kingdom	6 May 2022 to 24 August 2022	UK	European of Region (EUR)
France	19 May 2022 to 24 August 2022	FR	European of Region (EUR)
Canada	19 May 2022 to 24 August 2022	CA	Region of the Americas (AMR)

**Table 3:** Selecting the optimal ANN model with respect to single hidden layer and neurons for the USA dataset.

Items	Neurons	No. of Hidden Layers	Train		Validation		Test		Train-Rank		Validation-Rank		Test-Rank		Overall Score
			$R^{2}$	RMSE	$R^{2}$	RMSE	$R^{2}$	RMSE	$R^{2}$	RMSE	$R^{2}$	RMSE	$R^{2}$	RMSE	
1	1	1	0.99944	0.00664	0.995686	0.019571	0.850461	0.12549	6	6	12	11	14	16	65
2	2	1	0.99959	0.00571	0.996186	0.018451	0.853831	0.124226	8	7	16	15	23	20	89
3	3	1	0.99875	0.00993	0.996379	0.017769	0.84821	0.125533	3	3	21	20	7	14	68
4	4	1	0.99976	0.00429	0.996808	0.016497	0.849201	0.123598	17	17	24	24	10	21	113
5	5	1	0.99958	0.00570	0.996302	0.018165	0.851358	0.125562	7	8	18	19	17	13	82
6	6	1	0.99966	0.00519	0.99552	0.019826	0.850677	0.124633	11	11	7	8	15	18	70
7	7	1	0.99843	0.01092	0.996327	0.01819	0.856681	0.123359	2	2	19	18	24	24	89
8	8	1	0.99964	0.00529	0.995946	0.018947	0.852853	0.124228	10	10	13	14	20	19	86
9	9	1	0.99960	0.00558	0.996128	0.01833	0.847895	0.125273	9	9	15	17	6	17	73
10	10	1	0.99979	0.00407	0.995653	0.019472	0.847847	0.125773	18	18	11	12	5	11	75
11	11	1	0.99987	0.00317	0.996481	0.01761	0.848805	0.125995	23	23	22	22	9	10	109
12	12	1	0.99935	0.00718	0.995547	0.019842	0.853296	0.12359	4	4	8	7	22	22	67
13	13	1	0.99981	0.00389	0.994944	0.021484	0.84724	0.129037	22	22	6	6	4	6	66
14	14	1	0.99979	0.00403	0.996191	0.018423	0.850882	0.125673	20	20	17	16	16	12	101
15	15	1	0.9993	0.00699	0.995982	0.019144	0.852618	0.126268	5	5	14	13	19	8	64
16	16	1	0.99979	0.00406	0.996796	0.016956	0.850439	0.126403	19	19	23	23	13	7	104
17	17	1	0.99971	0.00474	0.992316	0.027267	0.852996	0.129848	15	15	2	2	21	4	59
18	18	1	0.99972	0.00472	0.995592	0.019731	0.848797	0.126132	16	16	9	9	8	9	67
19	19	1	0.99970	0.00481	0.99563	0.019641	0.849848	0.125492	13	13	10	10	12	15	73
**20**	**20**	**1**	**0.99980**	**0.00394**	**0.996354**	**0.01764**	**0.849419**	**0.123588**	**21**	**21**	**20**	**21**	**11**	**23**	**117**
21	21	1	0.99987	0.00314	0.993076	0.024351	0.83232	0.131779	24	24	4	4	1	2	59
22	22	1	0.99971	0.00475	0.994395	0.022199	0.841461	0.129095	14	14	5	5	3	5	46
23	23	1	0.99828	0.01138	0.987112	0.035651	0.852588	0.13128	1	1	1	1	18	3	25
24	24	1	0.99967	0.00508	0.99283	0.025737	0.840422	0.133195	12	12	3	3	2	1	33

**Table 4:** Selecting the optimal ANN model with respect to two hidden layer and neurons for the USA dataset.

Items	Neurons	No. of Hidden Layers	Train		Validation		Test		Train-Rank		Validation-Rank		Test-Rank		Overall Score
			$R^{2}$	RMSE	$R^{2}$	RMSE	$R^{2}$	RMSE	$R^{2}$	RMSE	$R^{2}$	RMSE	$R^{2}$	RMSE	
1	5	2	0.99958	0.0057	0.996302	0.018165	0.851358	0.125562	7	8	18	19	17	13	82
2	11	2	0.99987	0.00317	0.996481	0.01761	0.848805	0.125995	23	23	22	22	9	10	109
3	12	2	0.99935	0.00718	0.995547	0.019842	0.853296	0.12359	4	4	8	7	22	22	67
4	16	2	0.99979	0.00406	0.996796	0.016956	0.850439	0.126403	19	19	23	23	13	7	104
5	19	2	0.9997	0.00481	0.99563	0.019641	0.849848	0.125492	13	13	10	10	12	15	73

**Table 5:** Selecting the optimal ANN model with respect to hidden layers and neurons for the Germany dataset.

Items	Neurons	No. of Hidden Layers	Train		Validation		Test		Train-Rank		Validation-Rank		Test-Rank		Overall Score
			$R^{2}$	RMSE	$R^{2}$	RMSE	$R^{2}$	RMSE	$R^{2}$	RMSE	$R^{2}$	RMSE	$R^{2}$	RMSE	
1	7	1	0.99981	0.00506	0.998549	0.012344	0.960484	0.065853	18	18	12	12	16	16	92
2	15	1	0.99972	0.00604	0.998841	0.011029	0.959775	0.066482	9	9	16	17	12	10	73
3	20	1	0.99977	0.00554	0.998526	0.012422	0.961158	0.065168	11	11	11	11	19	19	82
4	22	1	0.99983	0.00476	0.997861	0.014981	0.962513	0.064003	21	21	2	2	22	23	91
**5**	**24**	**1**	**0.99993**	**0.00305**	**0.999307**	**0.008547**	**0.961561**	**0.06505**	**24**	**24**	**24**	**24**	**20**	**20**	**136**
6	5	2	0.99971	0.00633	0.998842	0.011072	0.960826	0.065823	7	7	17	16	17	17	81
7	10	2	0.99982	0.00487	0.999036	0.010025	0.960202	0.065854	20	20	23	23	15	15	116
8	11	2	0.99979	0.00532	0.99891	0.010686	0.959587	0.066564	16	16	19	19	9	9	88
9	12	2	0.99988	0.00395	0.998736	0.011531	0.962649	0.06398	23	23	14	15	23	24	122
10	21	2	0.99980	0.00509	0.998238	0.013561	0.959688	0.066353	17	17	7	7	11	13	72

**Table 6:** Selecting the optimal ANN model with respect to hidden layers and neurons for the UK dataset.

Items	Neurons	No. of Hidden layers	Train		Validation		Test		Train-Rank		Validation-Rank		Test-Rank		Overall Score
			$R^{2}$	RMSE	$R^{2}$	RMSE	$R^{2}$	RMSE	$R^{2}$	RMSE	$R^{2}$	RMSE	$R^{2}$	RMSE	
1	4	1	0.99616	0.0174	0.997668	0.011402	0.631966	0.223179	12	12	15	15	13	15	82
2	7	1	0.99613	0.01742	0.998503	0.009161	0.632303	0.222859	10	11	22	22	14	16	95
3	15	1	0.9964	0.01678	0.998149	0.010213	0.634408	0.221676	18	19	20	20	18	20	115
**4**	**18**	**1**	**0.99707**	**0.01515**	**0.998546**	**0.00899**	**0.631227**	**0.222802**	**22**	**23**	**24**	**24**	**12**	**17**	**122**
5	20	1	0.9971	0.01516	0.996542	0.014373	0.635039	0.227021	23	22	6	4	19	2	76
6	6	2	0.99596	0.01784	0.997928	0.010815	0.629863	0.225646	8	8	18	18	8	4	64
7	9	2	0.99614	0.01742	0.998515	0.009107	0.630533	0.224038	11	10	23	23	11	10	88
8	14	2	0.99647	0.01658	0.997121	0.012745	0.637717	0.218903	20	20	14	13	22	24	113
9	16	2	0.99639	0.01693	0.997793	0.011191	0.635548	0.222772	17	17	16	16	20	18	104
10	22	2	0.99629	0.01723	0.997893	0.011138	0.638272	0.223483	16	13	17	17	24	13	100

**Table 7:** Selecting the optimal ANN model with respect to hidden layers and neurons for the France dataset.

Items	Neurons	No. of Hidden layers	Train		Validation		Test		Train-Rank		Validation-Rank		Test-Rank		Overall Score
			$R^{2}$	RMSE	$R^{2}$	RMSE	$R^{2}$	RMSE	$R^{2}$	RMSE	$R^{2}$	RMSE	$R^{2}$	RMSE	
1	3	1	0.99437	0.02372	0.997974	0.016372	0.949289	0.08408	10	8	20	20	20	21	99
**2**	**5**	**1**	**0.99479**	**0.02259**	**0.998057**	**0.01597**	**0.948348**	**0.084555**	**15**	**15**	**22**	**22**	**18**	**17**	**109**
3	6	1	0.99435	0.02356	0.997897	0.016645	0.946519	0.086316	9	10	16	16	15	14	80
4	14	1	0.99442	0.02325	0.99691	0.019969	0.947747	0.084289	11	11	9	10	16	19	76
5	21	1	0.9952	0.02176	0.993804	0.02864	0.955583	0.078299	18	19	2	2	23	23	87
6	7	2	0.99465	0.02308	0.997937	0.016588	0.949004	0.084624	13	14	17	18	19	16	97
7	9	2	0.99452	0.02319	0.998034	0.016004	0.944522	0.087533	12	12	21	21	12	11	89
8	16	2	0.99525	0.02161	0.996716	0.020902	0.945894	0.087204	19	20	8	7	14	13	81
9	18	2	0.99604	0.01973	0.997475	0.018082	0.942191	0.089217	23	24	14	14	7	8	90
10	20	2	0.996	0.01982	0.997155	0.019208	0.94329	0.08837	22	22	11	11	10	10	86

**Table 8:** Selecting the optimal ANN model with respect to hidden layers and neurons for the Canada dataset.

Items	Neurons	No. of Hidden Layers	Train		Validation		Test		Train-Rank		Validation-Rank		Test-Rank		Overall Score
			$R^{2}$	RMSE	$R^{2}$	RMSE	$R^{2}$	RMSE	$R^{2}$	RMSE	$R^{2}$	RMSE	$R^{2}$	RMSE	
1	2	1	0.99754	0.01453	0.999267	0.010368	0.923376	0.110758	9	9	18	18	23	23	100
2	6	1	0.99743	0.01477	0.99944	0.009105	0.920556	0.113625	4	4	24	24	12	9	77
3	10	1	0.99756	0.01446	0.999316	0.010048	0.923644	0.110944	10	11	20	20	24	22	107
**4**	**16**	**1**	**0.99818**	**0.01248**	**0.999352**	**0.009834**	**0.921499**	**0.113232**	**19**	**19**	**23**	**23**	**19**	**13**	**116**
5	24	1	0.99844	0.01156	0.998198	0.016301	0.92087	0.112905	23	23	2	2	16	16	82
6	7	2	0.9976	0.01435	0.99933	0.009967	0.920149	0.113964	12	13	22	22	9	8	86
7	8	2	0.99781	0.0137	0.99915	0.011158	0.920551	0.112932	14	14	14	14	11	15	82
8	20	2	0.99789	0.01341	0.99915	0.011127	0.920659	0.112564	17	17	15	15	14	18	96
9	21	2	0.9984	0.01172	0.998421	0.015152	0.922865	0.110642	22	22	3	3	21	24	95
10	23	2	0.99848	0.01228	0.999123	0.011263	0.922201	0.110967	24	20	13	13	20	21	111

## Data Availability

The data used in this paper is available in the references in Section 2.7.
